# Using Field Data and GIS-Derived Variables to Model Occurrence of Williamson’s Sapsucker Nesting Habitat at Multiple Spatial Scales

**DOI:** 10.1371/journal.pone.0130849

**Published:** 2015-07-15

**Authors:** Mark C. Drever, Les W. Gyug, Jennifer Nielsen, A. Kari Stuart-Smith, I. Penny Ohanjanian, Kathy Martin

**Affiliations:** 1 Canadian Wildlife Service, Environment Canada, Delta, British Columbia, Canada; 2 Centre for Applied Conservation Research, Department of Forest and Conservation Sciences, University of British Columbia, Vancouver, British Columbia, Canada; 3 Okanagan Wildlife Consulting, West Kelowna, British Columbia, Canada; 4 Canadian Forest Products Ltd., Cranbrook, British Columbia, Canada; 5 Consulting Biologists, Kimberley, British Columbia, Canada; Institute of Zoology, CHINA

## Abstract

Williamson's sapsucker (*Sphyrapicus thyroideus*) is a migratory woodpecker that breeds in mixed coniferous forests in western North America. In Canada, the range of this woodpecker is restricted to three small populations in southern British Columbia, precipitating a national listing as ‘Endangered’ in 2005, and the need to characterize critical habitat for its survival and recovery. We compared habitat attributes between Williamson’s sapsucker nest territories and random points without nests or detections of this sapsucker as part of a resource selection analysis to identify the habitat features that best explain the probability of nest occurrence in two separate geographic regions in British Columbia. We compared the relative explanatory power of generalized linear models based on field-derived and Geographic Information System (GIS) data within both a 225 m and 800 m radius of a nest or random point. The model based on field-derived variables explained the most variation in nest occurrence in the Okanagan-East Kootenay Region, whereas nest occurrence was best explained by GIS information at the 800 m scale in the Western Region. Probability of nest occurrence was strongly tied to densities of potential nest trees, which included open forests with very large (diameter at breast height, DBH, ≥57.5 cm) western larch (*Larix occidentalis*) trees in the Okanagan-East Kootenay Region, and very large ponderosa pine (*Pinus ponderosa*) and large (DBH 17.5–57.5 cm) trembling aspen (*Populus tremuloides*) trees in the Western Region. Our results have the potential to guide identification and protection of critical habitat as required by the *Species at Risk Act* in Canada, and to better manage Williamson’s sapsucker habitat overall in North America. In particular, management should focus on the maintenance and recruitment of very large western larch and ponderosa pine trees.

## Introduction

Williamson’s sapsucker (*Sphyrapicus thyroideus*) is a migratory woodpecker that breeds in the montane mixed coniferous forests of western North America. Its breeding distribution extends from British Columbia to Baja California, Mexico [1]. In Canada, Williamson’s sapsucker is restricted to the southern interior of British Columbia where it occurs primarily in mid-elevation mixed coniferous forests containing either western larch (*Larix occidentalis*) or ponderosa pine (*Pinus ponderosa*), along with Douglas-fir (*Pseudotsuga menziesii*) and often containing a deciduous component of trembling aspen (*Populus tremuloides*) [2], [3]. In 2005, it was classified as ‘Endangered’ by the Committee on the Status of Endangered Wildlife in Canada, based primarily on low population numbers and projected rates of habitat loss, particularly for forests with mature western larch [4]. Consequently, identification and protection of Williamson’s sapsucker critical habitat is required for its conservation in Canada.

Habitat suitability models can help identify critical habitat, defined in the *Species at Risk Act* as ‘habitat that is necessary for the survival or recovery of a listed wildlife species’ [5]. These models can provide evidence for habitat attributes selected and used to a greater extent than suggested by their availability, and can thus be used to identify habitat features particularly important for management of species at risk. Sousa [6] developed a Habitat Suitability Index (HSI) for Williamson’s sapsuckers breeding in western North America, which involved four variables: percentage tree canopy closure, percentage of area dominated by aspen, average diameter at breast height (DBH) of overstory aspen, and number of suitable soft snags (DBH >30.5 cm, or DBH >45.7 cm for ponderosa pine) within a 4 hectare (ha) area around the nest [6]. When Sousa’s model was applied in Arizona [7], the HSI correctly indicated Williamson’s sapsucker selected nest territories in snow-melt drainages over areas on ridge tops, but incorrectly classified 63.6% of plots not used for nesting as optimal breeding habitat. Redefining variables and making the model region-specific may improve the degree of this misclassification.

Nesting habitat attributes used and selected by Williamson’s sapsucker appear to vary geographically. In Arizona, snag densities (i.e., number of dead trees) were the most important factor influencing nest location in stands dominated by aspen [7]. In Oregon, the sapsucker preferred to nest in forest areas with <75% canopy cover and <34 m^2^/ha basal area [8], with recommended estimates of 3.71 snags/ha with a DBH >30.5 cm to maximize a sapsucker population [9]. In British Columbia, densities of 20 trees/ha with a DBH >57 cm and 60–150 trees/ha with a DBH >22 cm were recommended to maintain Williamson’s sapsucker breeding habitat [10]. This variation indicates the sapsucker has flexible nesting requirements and may be selecting (or avoiding) particular habitat features in different systems.

Habitat selection by nesting Williamson’s sapsucker is likely influenced by both nesting and foraging requirements operating at multiple spatial scales from territories to the surrounding landscape. Crockett and Hadow [11] suggested nest trees in aspen were chosen based on proximity to foraging areas instead of nest tree features. Williamson’s sapsucker in Colorado had a minimum breeding territory size of 4 ha [12], while in British Columbia the minimum breeding territory was ~16 ha [10]. Territories must provide foraging opportunities for sapsuckers, including substantial quantities of sap and ants. Sap trees are typically coniferous trees (DBH 23–47 cm) within 100 m of the nest tree [13]. After eggs hatch, adults augment their diet with carpenter ants (*Camponotus* spp.) and other ants, which they also feed nestlings [14]. As carpenter ants depend on logs for nest substrate, the provision of downed logs within territories was considered an essential habitat attribute for nesting Williamson’s sapsucker [14]. While field measurements from the nest territory provide precise habitat use information at the territory scale, models derived from landscape-level attributes can be used to extrapolate probability of occurrence outside sampled areas [15]. Therefore, a comparison of habitat selection models using field- and GIS-derived variables may provide insights both into the scales at which Williamson’s sapsucker selects habitat attributes and into methods for characterizing habitat selection by the sapsucker.

In this paper, our primary objective was to identify variables influencing Williamson’s sapsucker nesting habitat selection at the spatial scale of breeding territories and, where possible, to identify associated threshold values. We performed a resource selection analysis [16], which compared habitat features at nest territories to areas where the sapsucker was confirmed to be absent (no detections of nests, sap feeding, or individuals), hereafter ‘random point’ or ‘no nest’. We used habitat measurements collected in the field and through a Geographic Information System (GIS) from the Vegetation Resource Inventory (VRI) databases maintained by the British Columbia Ministry of Forests, Lands and Natural Resource Operations. We compared the following sets of variables to assess which best explain nesting habitat selection: field measurements at 225 m radius and GIS information within a 225 m, 400 m, and 800 m radius of the nest. We then assessed the relative effectiveness of using available GIS databases to using field-derived data.

## Study Area

**Fig 1:**
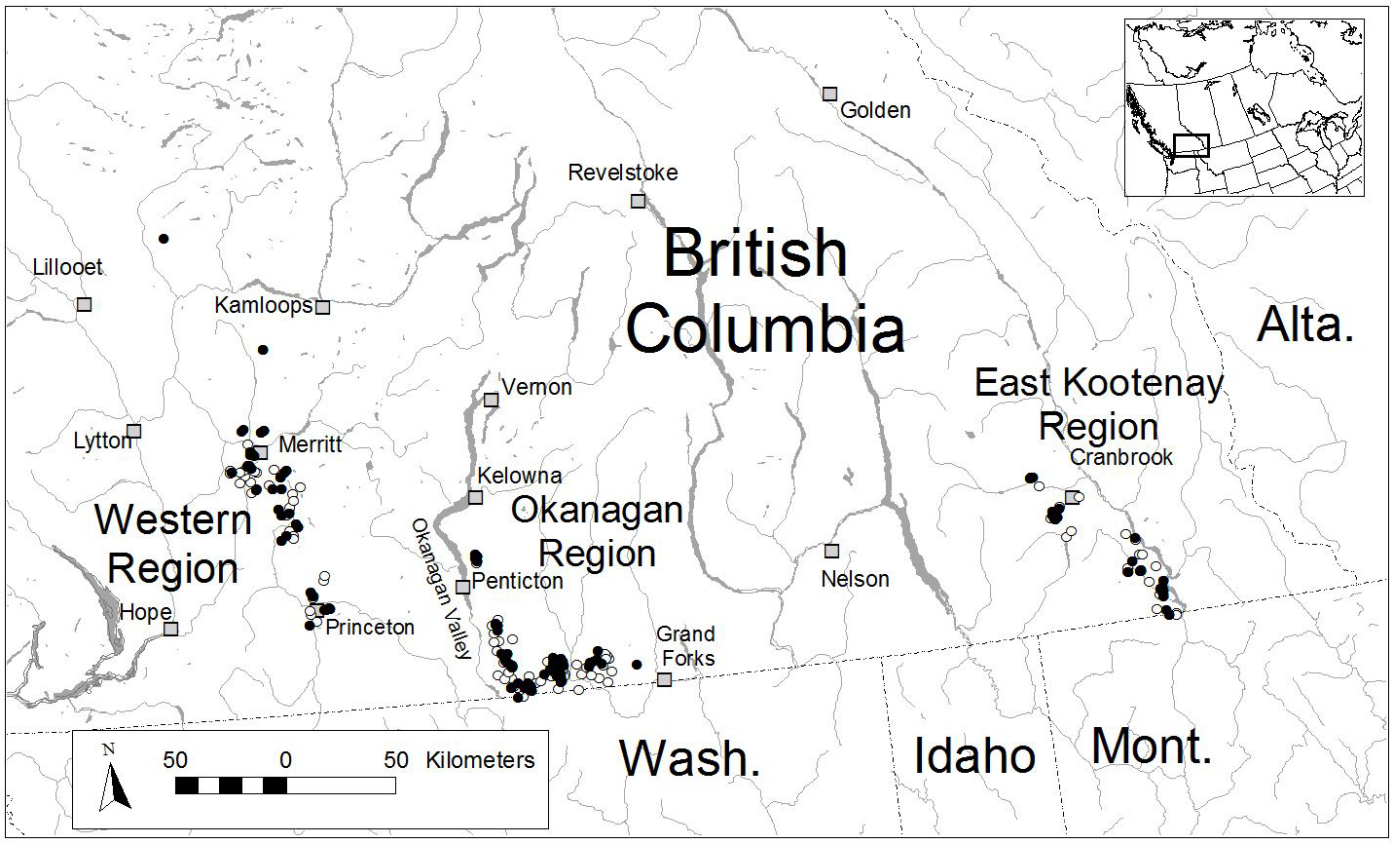
Map of the study area, indicating locations of Williamson’s sapsucker nests (black circles) and random points without detections (white circles) for three geographic regions where the species occurs in southern British Columbia, Canada.

This study encompassed the three disjunct geographic regions of the Williamson’s sapsucker breeding range in southern British Columbia [3]: the Western Region, west of the Okanagan valley principally near Merritt and Princeton; the Okanagan Region, east of Okanagan Lake and Okanagan Valley, south of Penticton and near the United States border; and the East Kootenay Region near Cranbrook (Fig 1). Forests cover much of the study area, dominated by Douglas-fir, western larch, ponderosa pine, lodgepole pine (*Pinus contorta*), trembling aspen, and hybrid spruce (*Picea glauca* x *engelmannii*). The forests of the Okanagan and East Kootenay Regions have similar species compositions, dominated by Douglas-fir and western larch; the Western Region is dominated by ponderosa pine and aspen, and lacking western larch. We therefore analyzed the Okanagan and East Kootenay Regions as one group (the Okanagan-East Kootenay Region), separate from the Western Region.

## Methods

### Ethics statement

The surveys were conducted primarily on public lands and private land with permission by landowner. No birds were handled and no samples collected. The Williamson’s Sapsucker Recovery Team (Canada) approved the data collection for avian nest and habitat monitoring. All surveys of nesting habitat were conducted to minimize disturbance on birds, usually after nesting activities were completed. All field activities were in agreement with federal and provincial legislation. Data used in this analysis can be freely accessed on the Government of Canada Open Data Portal (http://open.canada.ca/en/open-data) under the name ‘Williamson Sapsucker habitat plot data’.

### Field methods

We located Williamson's sapsucker nests during three field seasons (2006–2008) by using systematic call- and drum-playback (CPB) surveys to detect the presence of sapsuckers and later searching these areas for nests, following the methods in Gyug et al. [2]. In 2008, we chose random points within the area of known occupancy, and conducted 25–60 minute CPB surveys. We included at least five well-spaced CPB points within a 225 m radius of each accessible point in May-June to confirm the presence or absence of Williamson’s sapsucker [3].

We characterized forest habitat around nests and random points with no detection of Williamson’s sapsucker using circular vegetation plots in a 16 ha area (225 m radius). The 16 ha sampling area assumed a minimum territory size in which a breeding pair could meet its foraging requirements during the nesting period, based on mean nearest-neighbour nest distances of 450 m in the Okanagan-East Kootenay Region [2]. We used protocols from the Birds and Burns Network [17] for vegetation sampling of standing live and dead trees, with similar methods used by Nielsen-Pincus and Garton [18]. At each nest tree or plot center, we sampled a 50 m variable-width transect in each of the four cardinal directions, with north and south transects starting at the center and east and west transects starting 10 m from the center. In the remaining area around the nest or random point (60–225 m radius), we randomly sampled fifteen additional 50 m transects, with the constraint that they lay within the boundaries of forest stands mapped by the provincial Vegetation Resource Inventory (VRI). A random point within the 60–225 m radius was selected as the centre point for each random transect, and the orientation of each transect was determined by randomly selecting a bearing between 1 and 360 degrees.

Widths of sampling swaths across the centre line of a 50 m transect were specific to the DBH classes of trees: 2 m width for small trees (DBH 7.5–22.4 cm), 6 m width for large live trees (DBH 22.5–57.4 cm), and 20 m width for both large dead standing trees (‘snags’, DBH ≥22.5 cm) that were ≥1.4 m in height and for very large live trees (DBH ≥57.5 cm). These widths resulted in plot sizes of 0.01, 0.03 and 0.1 ha respectively, which we used to convert counts to stem densities (number per ha). We recorded the following for each tree within a plot: species, DBH within size classes outlined in British Columbia silvicultural guidelines (17.5–22.4, 22.5–37.4, 37.5–52.4, 52.5–57.4, 57.5–67.4, ≥67.5 cm) [19], tree condition (alive or dead), the presence of nest cavities, and if the tree was known to contain a Williamson's sapsucker cavity.

We counted downed logs along all 50 m transects, and measured their large end diameter (LED), length within the plot, and diameter at the large and small end within the plot. We determined downed log volume (m^3^/ha) using the plot method in 2006 and 2007 [20]. For most sites in 2007, we used the line intercept method [21] in addition to the plot method. Estimates of downed log volume calculated using the plot method and line method were strongly correlated (Pearson’s r = 0.93, N = 30, P < 0.001), so we averaged the values in 2007 for plots where both methods were applied. In 2008, we used only the line intercept method. We counted stumps if their diameter at stump height (DSH) was ≥22.5 cm.

### Geographic Information System (GIS) data

We extracted information from the Vegetation Resource Inventory (VRI) of the British Columbia Ministry of Forests and Range about vegetation cover within 225 m, 400 m and 800 m radius circles around the locations of nest trees and random points [22]. The VRI provided vegetation polygon characteristics derived from aerial photography calibrated with ground plots, from which we determined the total proportions of different habitat attributes. A preliminary evaluation revealed that the 400 m scale was strongly correlated with the 225 m scale (all Pearson’s r >0.85). Therefore, we only used the 225 m and 800 m radii in our analyses to capture the nest territory (i.e., minimum territory size for the sapsucker to meet breeding and foraging requirements) and an arbitrarily determined scale that was larger than this minimum territory scale. We defined habitat types by leading tree species, which included Douglas-fir, western larch, ponderosa pine, lodgepole pine, other coniferous species (total sum of hybrid spruce, subalpine fir, and other coniferous species observed), and broadleaf species, in each of four stand age classes based on available VRI information (0–39, 40–79, 80–119, and ≥120 yrs). In addition, we calculated the area consisting of cut blocks (<40 yrs old) in each polygon. We calculated two distance measures: the distance from a nest tree or random point to the nearest forest polygon aged ≥80 yrs (assigned a value of 0 if the point was in a forested polygon ≥80 yrs), and the distance from the nest or random point to the nearest edge of a forest cut block <40 yrs (assigned a value of 0 if the point was in a forest cut block <40 yrs).

### Data Analyses

We compiled a total of 211 initial field and GIS variables representing habitat attributes that may influence Williamson’s sapsucker nest territory selection (S1 Table). To examine whether and how the probability of Williamson’s sapsucker nest occurrence varied with these forest habitat features, we constructed a series of Generalized Linear Models (GLMs) with ‘nest’ or ‘no nest’ as a binary response variable (where nest territories received a value of 1, and areas with no nests a value of 0). We used habitat variables as explanatory variables in each GLM, with a binomial error distribution and logit link function [23].

We divided the measured explanatory variables into the Okanagan-East Kootenay Region (n = 166) and Western Region (n = 69) datasets, and removed habitat attributes with no variation. We screened these variables for correlations in a preliminary analysis, where we retained the more biologically relevant variable in a correlated pair (Pearson’s r >0.75) and excluded the other to avoid issues of multi-collinearity. This preliminary variable reduction resulted in 72 variables considered in model construction for the Okanagan-East Kootenay Region and 55 variables for the Western Region (Table 1).

**Table 1:** Numbers of explanatory variables considered in generalized linear models for the probability of Williamson’s sapsucker nest occurrence in two regions of southern British Columbia.

Variable type	Okanagan-East Kootenay Region					Western Region				
	Field225	GIS225	GIS800	GIS	Total	Field225	GIS225	GIS800	GIS	Total
Quadratic	4(19)	5(22)	3(12)	0(0)	12(17)	3(17)	4(25)	2(11)	0(0)	9(16)
Linear	6(29)	3(13)	3(12)	1(50)	13(18)	3(17)	4(25)	3(16)	0(0)	10(18)
Non-sig	11(52)	15(65)	20(77)	1(50)	47(65)	12(67)	8(50)	14(74)	2(100)	36(65)
Total	21	23	26	2	72	18	16	19	2	55

‘Quadratic’ indicates habitat variables were significant in a model that included a quadratic term; ‘Linear’ indicates habitat variables were significant as a linear term in the model; ‘Non-sig’ indicates habitat variable was not significant. Subheadings under each Region indicate the type of habitat variables: field measurements (‘Field225’), geographic information system (GIS) data from a 225 m radius around the nest or plot center (‘GIS225’), and GIS data from an 800 m radius around the nest or plot center (‘GIS800’). The two ‘GIS’ variables separated from the 225 m and 800 m scales are included in the modelling process for both scales. Numbers in brackets indicate the percent relative to the column total.

We first conducted an exploratory analysis that considered each habitat variable as an explanatory variable individually. We also fit a model including the quadratic term of each variable to allow for a non-linear relationship between the variable and the probability of nest occurrence. If this term was significant, we categorized the variable as ‘Quadratic’. Otherwise, we considered the variable as ‘Linear’ if the linear term was significant, or removed the variable from the final models if neither the quadratic nor linear terms were significant. We used a significance level of α = 0.1 to be conservative with our inclusion of variables, decrease the Type II error rate, and minimize the possibility of excluding potentially important habitat variables. We then tabulated the number of variables (per region) that were classified as Quadratic, Linear, or not significant (Table 1).

Following this initial evaluation, we assembled the significant variables in three separate models for each region: a ‘Field225’ model using field measurement data (10 variables in the Okanagan-East Kootenay Region, 6 variables in the Western Region), a ‘GIS225’ model using information within a 225 m radius of the nest or no nest plot center (9 variables in the Okanagan-East Kootenay Region, 8 variables in the Western Region), and a ‘GIS800’ model using information within an 800 m radius of the nest or no nest plot center (7 variables in the Okanagan-East Kootenay Region, 5 variables in the Western Region; Table 1). We removed variables that were not significant from these models to create the final six models from the remaining variables.

We statistically compared the final models within regions using Akaike Information Criterion corrected for small sample size (AICc) [24]. Scale parameters indicated that overdispersion was not an issue in any of the final models. For comparisons both within and between regions, we calculated area under the curve (AUC) values, and Hosmer-Lemeshow test statistics. We also assessed the effect of specific habitat variables using a graphical approach for inference. Using the range of observed values for the habitat variables, we obtained predictions and associated standard errors from the best model in which that habitat variable was included. We then plotted these predictions and their 95% prediction intervals together with observed data to evaluate the probability of nest territory occurrence. We used statistical packages from the program R (version 3.0.2) for all analyses [25], [26].

## Results

We measured habitat attributes at a total of 138 Williamson’s sapsucker nest territories (97 in the Okanagan-East Kootenay, 41 in the Western Region) and 96 random points with no detections of sapsucker presence or activity (68 in the Okanagan-East Kootenay, 28 in the Western Region).

The initial exploratory analyses indicated that, when considered singly, both field- and GIS-derived habitat variables could potentially explain variation in the probability of occurrence of Williamson’s sapsucker nests. Of the habitat variables considered in preliminary analyses, 25 of 72 (35%) in the Okanagan-East Kootenay Region, and 19 of 55 (35%) in Western Region were categorized as ‘Quadratic’ or ‘Linear’ (Table 1). When comparing field- versus GIS-derived variables, more field-derived variables (47%) tended to be significant relative to GIS-derived variables (25% at the 225 m and 36% at the 800 m scale) in the Okanagan-East Kootenay Region. In the Western Region, both types of variables had similar proportions classified as significant (34% of field variables, and 44% or 24% of GIS variables). The remaining variables were classified as not significant, and thus were not included in the secondary model construction.

The secondary model selection using the reduced suites of variables yielded a set of three models per region that all adequately fit the data, as indicated by the Hosmer-Lemeshow tests that had p-values >0.1 (Table 2). Rankings of models composed of field versus GIS-derived variables varied between the two regions. In the Okanagan-East Kootenay Region, the Field225 model best explained nest territory selection by Williamson’s sapsucker, having the lowest AICc value and highest AUC value. In the Western Region, the GIS800 model best explained variation in nest territory selection, having the lowest AICc value and highest AUC value. The GIS225 model had the poorest fit in each region. All final models provided reliable discrimination between nest territories and random points, with AUC values of ≥0.80 for all models in both regions, except the GIS225 model in the Okanagan-East Kootenay Region that had an AUC value of 0.72 (Table 2).

**Table 2:** Comparative statistics for models constructed of forest habitat variables in two regions of British Columbia.

Region	Variable type	AICc	AUC	Concordance (%)	Hosmer-Lemeshow test statistic(p-value)
Okanagan-East Kootenay Region	Field225	181.4	0.84	83.5	8.98(0.34)
	GIS225	209.5	0.73	72.3	9.11(0.33)
	GIS800	190.9	0.82	81.8	12.70(0.12)
Western Region	Field225	79.1	0.81	80.6	4.67(0.79)
	GIS225	80.1	0.80	79.8	10.63(0.22)
	GIS800	70.2	0.88	87.9	6.58(0.58)

For the Field225, GIS225, and GIS800 models in each region, comparative statistics are given: Akaike’s Information Criterion corrected for small sample sizes (AICc), area under the curve (AUC) of receiver operating characteristic, percentage of concordant pairs (Concordance) and the Hosmer-Lemeshow test statistic with the associated p-value.

**Fig 2:**
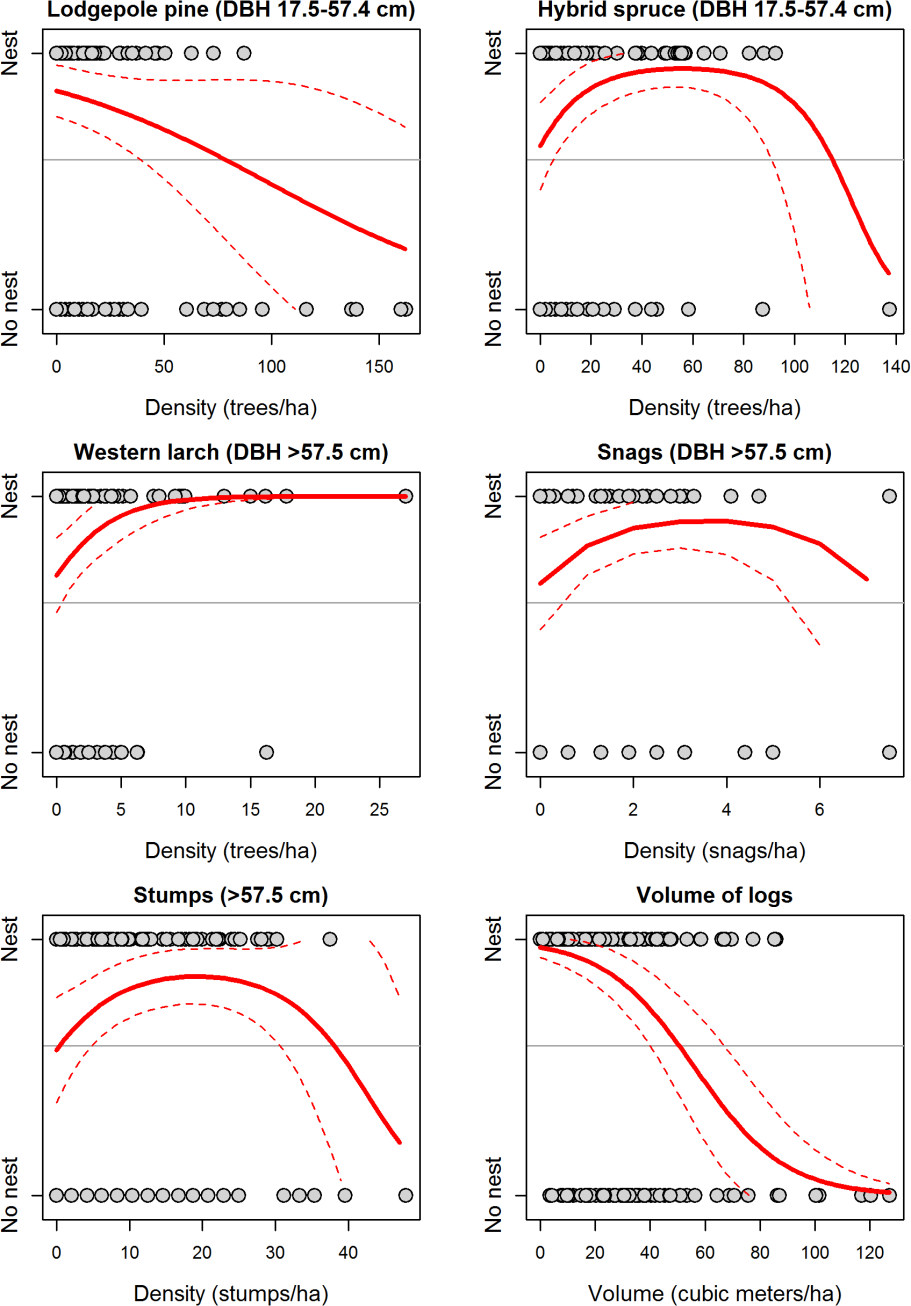
Predicted relationships between probability of nest occurrence and six forest habitat variables included in the Field225 model (21 variables originally considered) for the Okanagan-East Kootenay Region of the Williamson’s sapsucker Area of Occupancy in British Columbia, 2006–2008. Grey points depict nest occurrence (‘Nest’) or a random point (‘No nest’), and the title of each plot indicates the habitat variable. Solid lines indicate predicted values and thin dashed lines represent the 95% prediction interval. Horizontal line depicts proportion of points that were nests (i.e., the baseline probability of response variable having a value of 1).

Parameter values from each model indicated Williamson’s sapsucker nest territories were associated with habitat features related to suitable nest trees and foraging opportunities. In the Okanagan-East Kootenay Region, the Field225 model indicated Williamson’s sapsucker nest territories were positively associated with areas that had high densities of very large western larch trees (>8 trees/ha, DBH ≥57.5 cm), moderate densities of large hybrid spruce trees (50–70 trees/ha, DBH 17.5–57.4 cm), very large snags (4–5 snags/ha, DBH ≥57.5 cm), and very large stumps (18–20 stumps/ha, ≥57.5 cm diameter; Fig 2). Additionally, probability of nest occurrence was negatively associated with areas that had higher densities of large Douglas-fir and lodgepole pine trees (>25 trees/ha, DBH 17.5–57.4 cm) and higher volumes of downed logs (>15 m^3^/ha, LED ≥22.5 cm; Table 3, Fig 2).

**Table 3:** Parameter estimates for Field225, GIS225 and GIS800 models explaining Williamson’s sapsucker nest occurrence as a function of forest habitat variables in two regions of British Columbia.

Region	Variable type	Variable name	Coefficient	Standard error	z-score(p-value)
Okanagan-East Kootenay Region	Field225	Density of large live lodgepole pine trees (DBH 17.5–57.4 cm)	-0.018	0.009	-2.02(0.04)
	Field225	Density of large live hybrid spruce trees (DBH 17.5–57.4 cm)	0.078	0.028	2.76(0.006)
	Field225	Quadratic term of above variable	-0.001	0.0004	-1.90(0.06)
	Field225	Density of very large western larch (DBH ≥57.5 cm)	0.340	0.144	2.36(0.02)
	Field225	Density of very large snags (DBH ≥57.5)	0.896	0.355	2.53(0.01)
	Field225	Quadratic term of above variable	-0.126	0.063	-2.00(0.05)
	Field225	Density of very large stumps (DBH ≥57.5 cm)	0.156	0.061	2.56(0.01)
	Field225	Quadratic term of above variable	-0.004	0.002	-2.24(0.03)
	Field225	Volume of logs (LED ≥22.5 cm)	-0.061	0.014	-4.24(2.23×10^−05^)
	GIS225	Percent Douglas-fir 0–39 yrs (225 m)	-0.042	0.019	-2.17(0.03)
	GIS225	Percent Douglas-fir 40–79 yrs (225 m)	-0.029	0.015	-1.99(0.05)
	GIS225	Percent Douglas-fir 80–114 yrs (225 m)	0.018	0.008	2.27(0.02)
	GIS225	Mean area of canopy openings (225 m)	0.506	0.159	3.19(0.001)
	GIS225	Quadratic term of above variable	-0.039	0.013	-3.09(0.002)
	GIS800	Distance to forest polygon with logging history, age <40 yrs	-0.002	0.001	-2.21(0.03)
	GIS800	Percent Douglas-fir 0–39 yrs (800 m)	-0.163	0.052	-3.13(0.002)
	GIS800	Percent western larch 40–79 yrs (800 m)	-0.088	0.044	-2.00(0.05)
	GIS800	Quadratic term of above variable	0.001	0.001	1.82(0.07)
	GIS800	Percent western larch 80–119 yrs (800 m)	0.156	0.074	2.11(0.04)
	GIS800	Quadratic term of above variable	-0.007	0.003	-2.37(0.02)
	GIS800	Percent lodgepole pine 40–79 yrs (800 m)	-0.046	0.023	-1.99(0.05)
	GIS800	Percent lodgepole pine ≥120 yrs (800 m)	-0.965	0.314	-3.07(0.002)
	GIS800	Quadratic term of above variable	0.082	0.032	2.59(0.01)
Western Region	Field225	Density of large live trembling aspen trees (DBH 17.5–57.4 cm)	0.062	0.023	2.67(0.008)
	Field225	Density of very large ponderosa pine trees (DBH ≥57.5 cm)	1.183	0.409	2.89(0.004)
	Field225	Quadratic term of above variable	-0.134	0.064	-2.11(0.04)
	GIS225	Percent Douglas-fir ≥120 yrs (225 m)	-0.044	0.015	-3.01(0.003)
	GIS225	Mean crown closure of trees >15m in height (225 m)	0.276	0.091	3.05(0.002)
	GIS225	Quadratic term of above variable	-0.005	0.002	-3.07(0.002)
	GIS800	Percent non-forest vegetated area (800 m)	0.260	0.089	2.94(0.003)
	GIS800	Quadratic term of above variable	-0.006	0.002	-2.91(0.004)
	GIS800	Percent ponderosa pine 80–119 yrs (800 m)	0.100	0.045	2.20(0.03)
	GIS800	Percent ponderosa pine ≥120 yrs (800 m)	-0.237	0.123	-1.92(0.06)
	GIS800	Quadratic term of above variable	0.009	0.005	1.94(0.05)

Coefficients, standard errors of the coefficients, and z-score values are provided for each of the variables in the final models.

**Fig 3:**
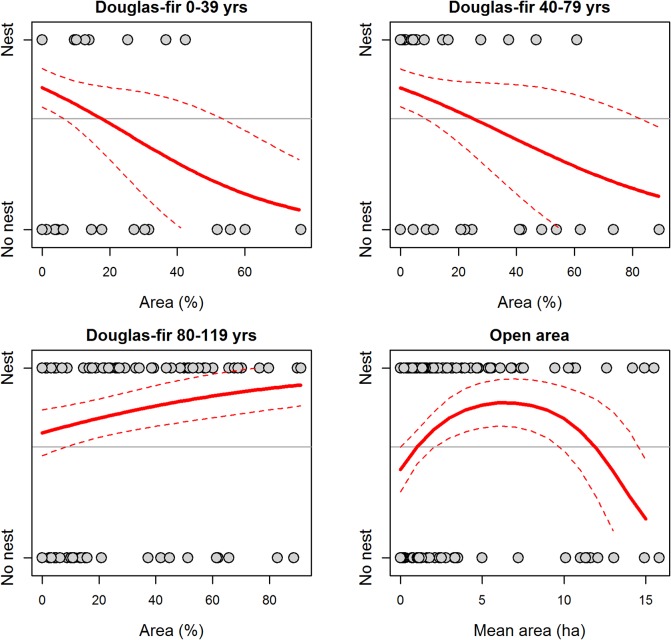
Predicted relationships between probability of nest occurrence and four forest habitat variables included in the GIS225 model (originally 25 variables considered) for the Okanagan-East Kootenay Region of the Williamson’s sapsucker Area of Occupancy in British Columbia, 2006–2008. Variables were calculated from geographic information system (GIS) habitat variables within a 225 m radius of the nest or random point. Grey points depict nest occurrence (‘Nest’) or a random point (‘No nest’), and the title of each plot indicates the habitat variable. Solid lines indicate predicted values and thin dashed lines represent the 95% prediction interval. Horizontal line depicts proportion of points that were nests (i.e., the baseline probability of response variable having a value of 1).

In the GIS225 model for the Okanagan-East Kootenay Region, probability of nest occurrence was positively associated with areas that had a high percentage (>80%) of older age classes of Douglas-fir trees (aged 80–119 yrs) in the canopy, and negatively associated with the percentage of canopy dominated by Douglas-fir trees in the younger age classes (< 80 yrs of age, Fig 3). In addition, this model indicated a non-linear relationship with the mean size of the openings around each nest or random point, where maximum probability of nest occurrence was in areas with openings averaging 6–7 ha (Fig 3).

**Fig 4:**
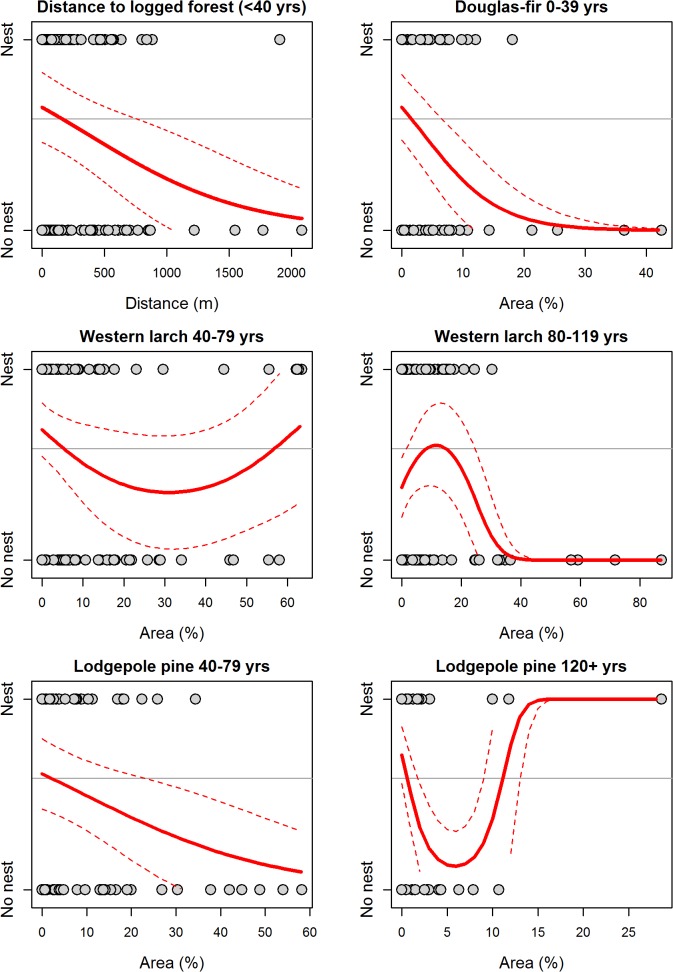
Predicted relationships between probability of nest occurrence and six forest habitat variables included in the GIS800 model (originally 28 variables considered) for the Okanagan-East Kootenay Region of the Williamson’s sapsucker Area of Occupancy in British Columbia, 2006–2008. Variables were calculated from geographic information system (GIS) data within an 800 m radius of the nest or random point. Grey points depict nest occurrence (‘Nest’) or a random point (‘No nest’), and the title of each plot indicates the habitat variable. Solid lines indicate predicted values and thin dashed lines represent the 95% prediction interval. Horizontal line depicts proportion of points that were nests (i.e., the baseline probability of response variable having a value of 1).

The GIS800 model for the Okanagan-East Kootenay Region indicated Williamson’s sapsucker selected nesting habitat relatively close to areas with a logging history in the last 40 yrs (<200 m, Fig 4). Nest occurrence in this model was negatively associated with a high percentage of Douglas-fir aged <40 yrs and lodgepole pine aged 40–79 yrs in the canopy (<2%, Fig 4). Nest occurrence was also negatively associated with areas having 5–7% canopy cover of lodgepole pine ≥120 yrs, or areas with 30% western larch aged 40–79 yrs, and was positively associated with areas having 10–15% western larch aged 80–119 yrs.

**Fig 5:**
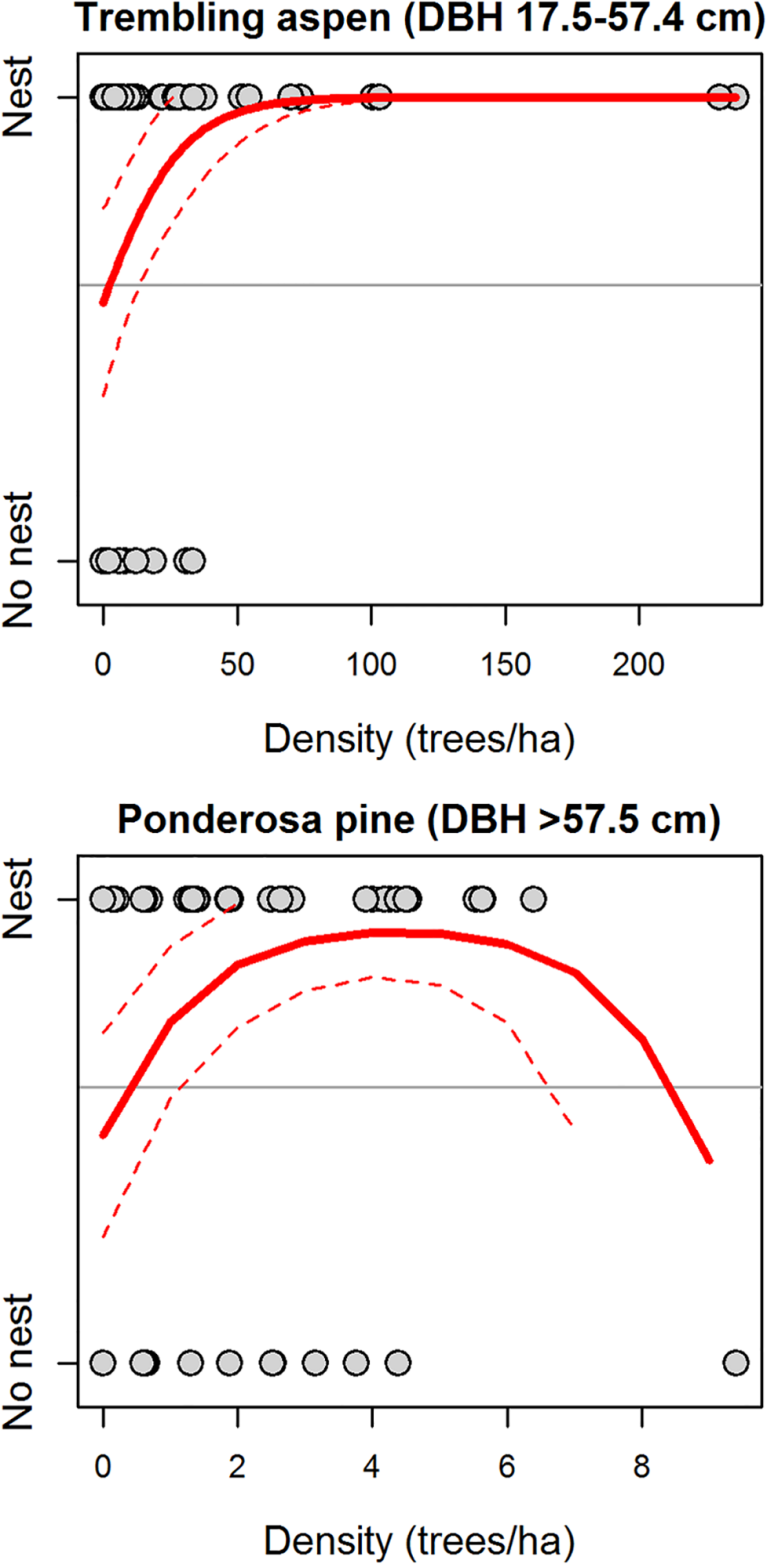
Predicted relationships between probability of nest occurrence and two forest habitat variables included in the Field225 model (18 variables originally considered) for the Western Region of the Williamson’s sapsucker Area of Occupancy in British Columbia, 2006–2008. Grey points depict nest occurrence (‘Nest’) or a random point (‘No nest’), and the title of each plot indicates the habitat variable. Solid lines indicate predicted values and thin dashed lines represent the 95% prediction interval. Horizontal line depicts proportion of points that were nests (i.e., the baseline probability of response variable having a value of 1).

**Fig 6:**
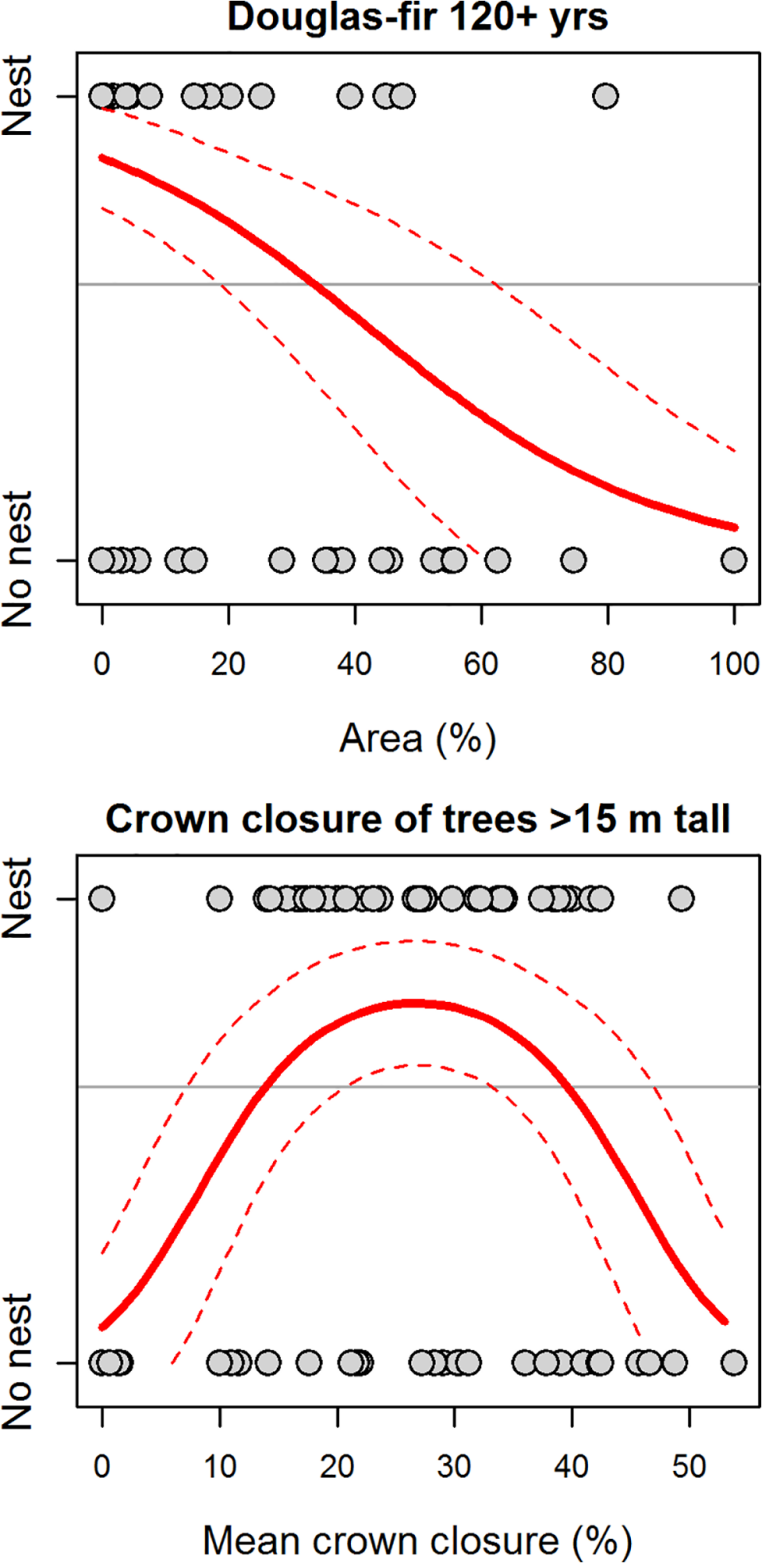
Predicted relationships between probability of nest occurrence and two forest habitat variables included in the GIS225 model (originally 18 variables considered) for the Western Region of the Williamson’s sapsucker Area of Occupancy in British Columbia, 2006–2008. Variables were calculated from geographic information system (GIS) data within a 225 m radius of the nest or random point. Grey points depict nest occurrence (‘Nest’) or a random point (‘No nest’), and the title of each plot indicates the habitat variable. Solid lines indicate predicted values and thin dashed lines represent the 95% prediction interval. Horizontal line depicts proportion of points that were nests (i.e., the baseline probability of response variable having a value of 1).

**Fig 7:**
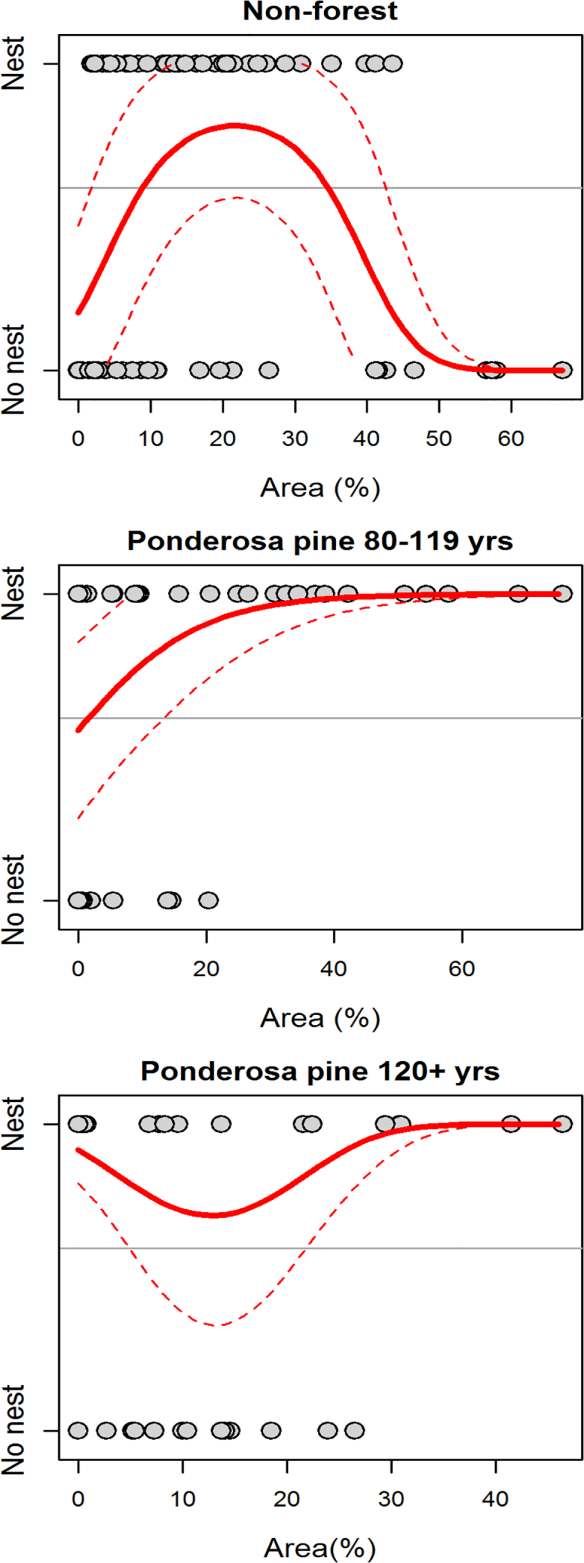
Predicted relationships between probability of nest occurrence and three forest habitat variables included in the GIS800 model (originally 21 variables considered) for the Western Region of the Williamson’s sapsucker Area of Occupancy in British Columbia, 2006–2008. Variables were calculated from geographic information system (GIS) data within an 800 m radius of the nest or random point. Grey points depict nest occurrence (‘Nest’) or a random point (‘No nest’), and the title of each plot indicates the habitat variable. Solid lines indicate predicted values and thin dashed lines represent the 95% prediction interval. Horizontal line depicts proportion of points that were nests (i.e., the baseline probability of response variable having a value of 1).

In the Western Region, models suggested Williamson’s sapsucker selected moderately-open habitats with complex relationships with older age classes of ponderosa pine trees. In the Field225 model, the probability of Williamson’s sapsucker nest occurrence was positively related to higher densities of large trembling aspen (>50 trees/ha, DBH 17.5–57.4 cm) and moderate densities of very large ponderosa pine trees (4–5 trees/ha, DBH ≥57.5 cm; Table 3, Fig 5). The GIS225 model indicated nest occurrence was negatively associated with a high percentage of Douglas-fir trees ≥120 yrs old (>10%), and positively associated with moderate amounts of crown closure (25–30%, >15 m tall; Fig 6). In the GIS800 model, nest occurrence was positively associated with 20–25% of an area covered by non-forest, positively associated with higher percentages of ponderosa pine aged 80–119 yrs in the canopy (>25%), and negatively associated with areas that had 10–15% of ponderosa pine aged ≥120 yrs in the canopy, suggesting a threshold relationship with crown closure (Fig 7).

## Discussion

The habitat features associated with the presence of Williamson’s sapsucker nests varied between our two study regions with respect to the relative utility of field versus GIS-derived habitat variables for modelling probability of occurrence. In the Okanagan-East Kootenay Region, nest territories tended to occur in areas that contained open mature forests with very large western larch trees, with the model derived from field variables having the best predictive performance. Western larch typically occurs in mixed stands with other conifers rather than in pure stands, and therefore its occurrence may be better quantified by field surveys than by interpreted aerial photos. In contrast, nest territories in the Western Region occurred in forests with high densities of large trembling aspen trees and very large ponderosa pine trees, both of which can occur in pure stands, and thus may be well characterized from aerial photos, resulting in the better predictive performance of GIS800 model in this region. The good predictive performance of this model in the Western Region suggests Williamson’s sapsuckers may have larger territories here than in other regions of British Columbia, as suggested by Gyug et al. [2]. However, the GIS-based models should be validated with an independent dataset before management recommendations for the Western Region are made without consideration of field measurements, as other underlying processes may also differ between the two regions.

Williamson’s sapsucker generally selects areas with relatively high densities of suitable nesting trees [6], [8], [11], [27]. In the Okanagan-East Kootenay Region, the sapsucker selected areas with high densities of very large (DBH >57.5 cm) dead trees and very large live western larch trees. In this region, distribution of western larch may be a limiting factor for nesting habitat selection [10]. Very large western larch trees are high quality nesting trees that provide an excellent substrate for excavation as their heartwood is often decayed and surrounded by decay-resistant sapwood, which may provide greater cavity stability and offer protection from nest predators such as squirrels, weasels, and bears [27], [28]. Very large western larch trees can stand for centuries [29], and may thus provide long-term breeding sites for Williamson’s sapsucker, a species known to reuse nest trees for multiple years. The western larch forests in British Columbia have the highest known breeding density of Williamson’s sapsucker (3.1 nests/km^2^) [2]. Therefore, management of Williamson’s sapsucker in the Okanagan-East Kootenay Region should focus on retention and recruitment of very large western larch trees.

Williamson’s sapsucker nests occurred in areas with relatively low volumes of coarse woody debris in the Okanagan-East Kootenay Region. Nielsen-Pincus and Garton [18] also found that nest sites of Williamson’s sapsucker had lower densities of downed logs than random points. Gyug et al. [14] suggested that dead and decaying wood should be managed to support ant colonies, which provide a major food source for Williamson’s sapsucker during the breeding season. This results in an apparent conundrum, as Williamson’s sapsucker selected breeding territories with a reduced volume of downed logs, but more logs should result in higher numbers of ant colonies, which should be correlated with higher preference, if preference was based on prey abundance alone. There may be a threshold value of downed logs above which higher densities of ants become superfluous to the breeding requirements of Williamson’s sapsucker, or there may be reservoirs of ants in other types of dead and decaying wood. The Okanagan-East Kootenay region experiences a mixed-severity fire regime [30], so Williamson’s sapsucker may select for open stands of western larch historically maintained by frequent ground fires, which would have consumed most logs on the forest floor [29], [31]. Alternatively, the selection of very large trees may result in sapsuckers using older forest areas where log volumes have degraded over time.

Nesting territories were positively associated with the presence of ponderosa pine trees in the Western Region, where measures of large diameter trees and older age classes of ponderosa pine were significant explanatory variables in both the GIS800 and Field225 models. Williamson’s sapsucker excavate cavities in ponderosa pine trees [10], although it most commonly nests in trembling aspen where suitable western larch trees are unavailable [1], [11]. A relatively high density of large trembling aspen trees (>50 trees/ha, DBH 17.5–57.4 cm) was also positively associated with Williamson’s sapsucker nest territories in the Western Region. The probability of using aspen as a nesting tree differs throughout the Williamson’s sapsucker range; where present and abundant, aspen was often a significant nest tree species, but where absent, suitable conifers were used [10]. Therefore, Williamson’s sapsucker habitat selection models should be developed to accommodate the variable distribution of tree species across its range.

Under the *Species at Risk Act*, government agencies in Canada have a legislated responsibility to identify and protect critical habitat of species at risk, and implement a Recovery Strategy or Action Plan. Our results can be used to inform this process in two ways. First, models can be validated and then used in combination with previous habitat suitability models [15] to map locations of critical habitat for Williamson’s sapsucker. Mapping the relevant GIS-derived variables may be complicated by the nature of the VRI data, which were derived for timber volume estimation based on the interpretation of aerial photographs. For example, habitat features that predict a high probability of Williamson’s sapsucker nest occurrence, such as densities of very large western larch, may not be mapped if patches are too small (i.e., <3 ha), or if these trees contribute little to merchantable stand volume. Therefore, delineation of critical habitat is likely best identified by spatial analyses that combine field- and GIS-derived variables, as in Sousa [6] and as currently proposed in the recovery strategy for Williamson’s sapsucker [15]. Second, important habitat features identified from field measurement data can guide specific management actions to ensure their retention and effective protection on the landscape.

Retention of large diameter western larch within Williamson’s sapsucker habitat in the Okanagan-East Kootenay Region should be a key component of management plans. Long-term management should involve recruitment of large diameter western larch, perhaps by reducing stem density of small diameter trees (‘thinning-from-below’), given that western larch responds well to thinning operations [32]. No variables related to sap trees (i.e., small diameter Douglas-fir) were significant with nest territory selection by Williamson’s sapsucker, so these thinning operations would not appear to affect foraging requirements of the species. Prescribed burns would also reduce small diameter stem densities, clear the understory, and mimic the natural disturbance regime that likely created these open, mature stands. A combination of thinning and prescribed under-burns showed promise for successfully restoring an old-growth western larch forest in Montana [33]. Such an approach would be extremely valuable for restoration efforts aimed at increasing overall available habitat to increase populations of Williamson’s sapsuckers in regions where habitat is limited. Future research should seek to validate the habitat selection models presented here by manipulation of stem density or other experiments that may identify management practices useful in improving habitat quality and augmenting Williamson’s sapsucker populations.

Similar considerations should be made for aspen stands within ponderosa pine forests. Our findings in the Western Region support the well-established relationship and biological importance of large diameter trembling aspen to Williamson’s sapsucker and the broader cavity nester guild [1], [10], [11], [34]. Old-growth western larch and aspen stands with large diameter trees provide valuable habitat for many bird species in the area [35], [36]. Thus, conservation efforts for Williamson’s sapsucker will likely have collateral benefits for avian biodiversity in mature conifer and mixed forests as a whole.

## Supporting Information

S1 Table Habitat variables (211 variables) considered in the construction of Williamson’s sapsucker nest territory selection models, prior to variable reduction. All GIS variables are repeated for the 225 m, 400 m and 800 m scales, with the exceptions of CROWNGT15_225, MaxCRNGT15225, MNCRN15225, DIST_TO_AGE_80 and DIST_to_OPEN_LT40yrs.
(DOCX)
