# Supplementary material for: Using Field Data and GIS-Derived Variables to Model Occurrence of Williamson’s Sapsucker Nesting Habitat at Multiple Spatial Scales
Source: PLoS One. 2015 Jul 15;10(7):e0130849. doi: 10.1371/journal.pone.0130849 (PMC4503628; doi:10.1371/journal.pone.0130849)
Supplement: S1 Table — All GIS variables are repeated for the 225 m, 400 m and 800 m scales, with the exceptions of CROWNGT15_225, MaxCRNGT15225, MNCRN15225, DIST_TO_AGE_80 and DIST_to_OPEN_LT40yrs. (DOCX) [file pone.0130849.s001.docx]

**S1 Table. Habitat variables (211 variables) considered in the construction of Williamson’s sapsucker nest territory selection models, prior to variable reduction.** All GIS variables are repeated for the 225m, 400m and 800m scales, with the exceptions of CROWNGT15_225, MaxCRNGT15225, MNCRN15225, DIST_TO_AGE_80 and DIST_to_OPEN_LT40yrs.

| Variable code | Variable type | Variable name |
| --- | --- | --- |
| D_LT_S | Field | Density of small live trees (DBH 7.5-17.4 cm) |
| D_LT_L | Field | Density of large live trees (DBH 17.5-57.4 cm) |
| D_LT_VL | Field | Density of very large live trees (DBH ≥57.5 cm) |
| D_LT | Field | Density of merchantable live trees (DBH ≥17.5 cm) |
| D_LT_GT8 | Field | Density of all live trees (DBH ≥7.5 cm) |
| D_SN_L | Field | Density of large snags (22.5-57.4 cm) |
| D_SN_VL | Field | Density of very large snags (≥57.5 cm) |
| D_SN_ALL | Field | Density of all snags (≥22.5 cm) |
| D_ST_L | Field | Density of large stumps (22.5-57.4 cm) |
| D_ST_VL | Field | Density of very large stumps (≥57.5 cm) |
| D_ST_ALL | Field | Density of all stumps (≥22.5 cm) |
| D_VL | Field | Density of very large trees, snags, and stumps (≥57.5 cm) |
| STUMPS | Field | Cut stumps present (≥22.5 cm) |
| D_LT_10 | Field | Density of live trees (DBH <12.5 cm) |
| D_LT_15 | Field | Density of live trees (DBH 12.5-17.4 cm) |
| D_LT_20 | Field | Density of live trees (DBH 17.5-22.4 cm) |
| D_LT_25 | Field | Density of live trees (DBH 22.5-27.4 cm) |
| D_LT_30 | Field | Density of live trees (DBH 27.5-32.4 cm) |
| D_LT_35 | Field | Density of live trees (DBH 32.5-37.4 cm) |
| D_LT_40 | Field | Density of live trees (DBH 37.5-42.4 cm) |
| D_LT_45 | Field | Density of live trees (DBH 42.5-47.4 cm) |
| D_LT_50 | Field | Density of live trees (DBH 47.5-52.4 cm) |
| D_LT_55 | Field | Density of live trees (DBH 52.5-57.4 cm) |
| D_LT_60 | Field | Density of live trees (DBH 57.5-62.4 cm) |
| D_LT_65 | Field | Density of live trees (DBH 62.5-67.4 cm) |
| D_LT_70 | Field | Density of live trees (DBH ≥67.5 cm) |
| P_LT_At | Field | Percent live trembling aspen trees (DBH ≥17.5 cm) |
| P_LT_OT | Field | Percent other species of live trees (DBH ≥17.5 cm) |
| P_LT_Fd | Field | Percent live Douglas-fir trees (DBH ≥17.5 cm) |
| P_LT_Lw | Field | Percent live western larch trees (DBH ≥17.5 cm) |
| P_LT_Pl | Field | Percent live lodgepole pine trees (DBH ≥17.5 cm) |
| P_LT_Py | Field | Percent live ponderosa pine trees (DBH ≥17.5 cm) |
| P_LT_S | Field | Percent live hybrid spruce trees (DBH ≥17.5 cm) |
| P_LT_B | Field | Percent live subalpine fir trees (DBH ≥17.5 cm) |
| P_LT_FdLw | Field | Percent live Douglas-fir and western larch trees (DBH ≥17.5 cm) |
| P_LT_FAPy | Field | Percent live Douglas-fir, trembling aspen, and ponderosa pine trees (DBH ≥17.5 cm) |
| P_LT_APO | Field | Percent live trembling aspen, ponderosa pine, and other tree species (DBH ≥17.5 cm) |
| P_LT_SPB | Field | Percent live hybrid spruce, lodgepole pine, and subalpine fir trees (DBH ≥17.5 cm) |
| P_LT_L_At | Field | Percent large live trembling aspen trees (DBH 17.5-57.4 cm) |
| P_LT_L_OT | Field | Percent other species of large live trees (DBH 17.5-57.4 cm) |
| P_LT_L_Fd | Field | Percent large live Douglas-fir trees (DBH 17.5-57.4 cm) |
| P_LT_L_Lw | Field | Percent large live western larch trees (DBH 17.5-57.4 cm) |
| P_LT_L_Pl | Field | Percent large live lodgepole pine trees (DBH 17.5-57.4 cm) |
| P_LT_L_Py | Field | Percent large live ponderosa pine trees (DBH 17.5-57.4 cm) |
| P_LT_L_S | Field | Percent large live hybrid spruce trees (DBH 17.5-57.4 cm) |
| P_LT_L_B | Field | Percent large live subalpine fir trees (DBH 17.5-57.4 cm) |
| P_LT_VL_At | Field | Percent very large live trembling aspen trees (DBH ≥57.5 cm) |
| P_LT_VL_OT | Field | Percent other species of very large live trees (DBH ≥57.5 cm) |
| P_LT_VL_Fd | Field | Percent very large live Douglas-fir trees (DBH ≥57.5 cm) |
| P_LT_VL_Lw | Field | Percent very large live western larch trees (DBH ≥57.5 cm) |
| P_LT_VL_Pl | Field | Percent very large live lodgepole pine trees (DBH ≥57.5 cm) |
| P_LT_VL_Py | Field | Percent very large live ponderosa pine trees (DBH ≥57.5 cm) |
| P_LT_VL_S | Field | Percent very large live hybrid spruce trees (DBH ≥57.5 cm) |
| P_LT_VL_B | Field | Percent very large live subalpine fir trees (DBH ≥57.5 cm) |
| D_CAVW_NP | Field | Density of possible nest trees in the nest patch (no./ha) |
| D_CAVW_REST | Field | Density of possible nest trees in the remaining 15 ha (no./ha) |
| D_CAVW_SUM | Field | Density of possible nest trees in 16 ha (no./ha) |
| D_CAV_WTOT | Field | Density of total possible + nest tree (0.0625/ha) |
| D_CAV_NON | Field | Density of trees or snags with cavities but not suitable for Williamson's sapsucker (no./ha) |
| D_CAV_ALL | Field | Density of all trees or snags with cavities not including nest tree (0.0625/ha) |
| D_CAV_TOT | Field | Density of all trees or snags with cavities including nest tree (0.0625/ha) |
| D_LG_ALL | Field | Density of all logs (no./ha, LED ≥22.5 cm) |
| D_LG_L | Field | Density of large logs (no./ha, LED 22.5-57.4 cm) |
| D_LG_VL | Field | Density of very large logs (no./ha, LED ≥57.5 cm) |
| V_LG_S | Field | Volume of small logs (m3/ha, LED 7.5-22.4 cm) |
| V_LG_PT | Field | Log volume using plot method (m3/ha, LED ≥22.5 cm) |
| V_LG_IN | Field | Log volume using intercept method (m3/ha, LED ≥22.5 cm) |
| V_LG_MN | Field | Log volume using mean of plot and intercept methods (m3/ha, LED ≥22.5 cm) |
| V_LG_L | Field | Volume of large logs (m3/ha, LED 22.5-57.4 cm) |
| V_LG_VL | Field | Volume of very large logs (m3/ha, LED ≥57.5 cm) |
| P_LG_Fd | Field | Percent Douglas-fir in log volume |
| P_LG_Lw | Field | Percent western larch in log volume |
| P_LG_Py | Field | Percent ponderosa pine in log volume |
| P_LG_OT | Field | Percent other tree species in log volume |
| D_LT_At_GT37_5 | Field | Density of aspen suitable for nesting (DBH >37.4 cm) |
| D_LT_L_Fd | Field | Density large live Douglas-fir trees (no./ha, DBH 17.5-57.4 cm) |
| D_LT_L_othersap | Field | Density of large coniferous trees as putative sap trees (no./ha, DBH 17.5-57.4 cm) |
| D_LT_L_Lw | Field | Density large live western larch trees (no./ha, DBH 17.5-57.4 cm) |
| D_LT_L_At | Field | Density large live trembling aspen trees (no./ha, DBH 17.5-57.4 cm) |
| D_LT_L_Pl | Field | Density large live lodgepole pine trees (no./ha, DBH 17.5-57.4 cm) |
| D_LT_L_Py | Field | Density large live ponderosa pine trees (no./ha, DBH 17.5-57.4 cm) |
| D_LT_L_S | Field | Density large live hybrid spruce trees (no./ha, DBH 17.5-57.4 cm) |
| D_LT_L_B | Field | Density large live subalpine fir trees (no./ha, DBH 17.5-57.4 cm) |
| D_LT_L_OT | Field | Density other species of large live trees (no./ha, DBH 17.5-57.4 cm) |
| D_LT_conif | Field | Density live coniferous trees (no./ha, DBH ≥17.5 cm) |
| D_LT_Lw | Field | Density live western larch trees (no./ha, DBH ≥17.5 cm) |
| D_LT_At | Field | Density live trembling aspen trees (no./ha, DBH ≥17.5 cm) |
| D_LT_Fd | Field | Density live Douglas-fir trees (no./ha, DBH ≥17.5 cm) |
| D_LT_Pl | Field | Density live lodgepole pine trees (no./ha, DBH ≥17.5 cm) |
| D_LT_Py | Field | Density live ponderosa pine trees (no./ha, DBH ≥17.5 cm) |
| D_LT_S | Field | Density live hybrid spruce trees (no./ha, DBH ≥17.5 cm) |
| D_LT_B | Field | Density live subalpine fir trees (no./ha, DBH ≥17.5 cm) |
| D_LT_VL_conif | Field | Density very large live coniferous trees (no./ha, DBH ≥57.5 cm) |
| D_LT_VL_Lw | Field | Density very large live western larch trees (no./ha, DBH ≥57.5 cm) |
| D_LT_VL_At | Field | Density very large live trembling aspen trees (no./ha, DBH ≥57.5 cm) |
| D_LT_VL_Fd | Field | Density very large live Douglas-fir trees (no./ha, DBH ≥57.5 cm) |
| D_LT_VL_Pl | Field | Density very large live lodgepole pine trees (no./ha, DBH ≥57.5 cm) |
| D_LT_VL_Py | Field | Density very large live ponderosa pine trees (no./ha, DBH ≥57.5 cm) |
| D_LT_VL_S | Field | Density very large live hybrid spruce trees (no./ha, DBH ≥57.5 cm) |
| D_LT_VL_B | Field | Density very large live subalpine fir trees (no./ha, DBH ≥57.5 cm) |
| *GIS variables (repeated at the 225m, 400m and 800m scales)* | | |
| Fd_A_225 | GIS | Percent Douglas-fir leading 0-39 yrs |
| Fd_B_225 | GIS | Percent Douglas-fir leading 40-79 yrs |
| Fd_C_225 | GIS | Percent Douglas-fir leading 80-119 yrs |
| Fd_D_225 | GIS | Percent Douglas-fir leading ≥120 yrs |
| Lw_A_225 | GIS | Percent western larch leading 0-39 yrs |
| Lw_B_225 | GIS | Percent western larch leading 40-79 yrs |
| Lw_C_225 | GIS | Percent western larch leading 80-119 yrs |
| Lw_D_225 | GIS | Percent western larch leading ≥120 yrs |
| Py_A_225 | GIS | Percent ponderosa pine leading 0-39 yrs |
| Py_B_225 | GIS | Percent ponderosa pine leading 40-79 yrs |
| Py_C_225 | GIS | Percent ponderosa pine leading 80-119 yrs |
| Py_D_225 | GIS | Percent ponderosa pine leading ≥120 yrs |
| Pl_A_225 | GIS | Percent lodgepole pine leading 0-39 yrs |
| Pl_B_225 | GIS | Percent lodgepole pine leading 40-79 yrs |
| Pl_C_225 | GIS | Percent lodgepole pine leading 80-119 yrs |
| Pl_D_225 | GIS | Percent lodgepole pine leading ≥120 yrs |
| Conif_A_225 | GIS | Percent other coniferous tree species leading 0-39 yrs |
| Conif_B_225 | GIS | Percent other coniferous tree species leading 40-79 yrs |
| Conif_C_225 | GIS | Percent other coniferous tree species leading 80-119 yrs |
| Conif_D_225 | GIS | Percent other coniferous tree species leading ≥120 yrs |
| Broadleaf_A_225 | GIS | Percent broadleaf tree species leading 0-39 yrs |
| Broadleaf_B_225 | GIS | Percent broadleaf tree species leading 40-79 yrs |
| Broadleaf_C_225 | GIS | Percent broadleaf tree species leading 80-119 yrs |
| Broadleaf_D_225 | GIS | Percent broadleaf tree species leading ≥120 yrs |
| Nospecies_A_225 | GIS | No tree species present |
| Nonforest_A_225 | GIS | Non-forest area |
| Fd_225 | GIS | Percent Douglas-fir, all age classes |
| Lw_225 | GIS | Percent western larch, all age classes |
| Py_225 | GIS | Percent ponderosa pine, all age classes |
| Pl_225 | GIS | Percent lodgepole pine, all age classes |
| Conif_225 | GIS | Percent other coniferous tree species, all age classes |
| Broadleaf_225 | GIS | Percent broadleaf tree species, all age classes |
| All.other.Conif_D_225 | GIS | Percent coniferous tree species other than western larch leading ≥120 yrs |
| Othersap.Conif_C_225 | GIS | Percent coniferous tree species other than Douglas-fir leading 80-119 yrs |
| Broadleaf_CD_225 | GIS | Percent broadleaf tree species leading ≥80 yrs |
| mean.openHA_225 | GIS | Mean area of openings in canopy |
| tot.opening_225 | GIS | Total area of openings in canopy |
| n.openings_225 | GIS | Number of openings in canopy |
| *GIS variables not repeated for different scales* | | |
| CROWNGT15_225 | GIS | Percentage crown closure of trees >15 m in height at 225 m scale |
| MaxCRNGT15225 | GIS | Maximum crown closure of trees >15 m in height at 225 m scale |
| MNCRN15225 | GIS | Mean crown closure of trees >15 m in height at 225 m scale |
| DIST_TO_AGE_80 | GIS | Distance to forest cover polygon of age ≥80 (m) |
| DIST_to_OPEN_LT40yrs | GIS | Distance to forest cover polygon with logging history and age <40 yrs (m) |
